# Ultrasound-Guided Lateral Femoral Cutaneous Nerve Block in Patients with Meralgia Paresthetica: Technical Description and Case Series of 11 Patients

**DOI:** 10.3390/jcm15083094

**Published:** 2026-04-18

**Authors:** Bosco Baron-Franco, Luis Beltran-Romero, Carlos Jiménez-Juan, Dolores Nieto-Martin, Santiago Rodriguez-Suarez, Maximo Bernabeu-Wittel

**Affiliations:** 1 Internal Medicine Department, University Hospital Virgen del Rocío, 41013 Seville, Spain; 2 Department of Medicine, University of Seville, 41004 Seville, Spain

**Keywords:** pain, ultrasound-guided, meralgia paresthetica, internal medicine

## Abstract

**Background**: Meralgia paresthetica is a neuropathic pain syndrome caused by compression of the lateral femoral cutaneous nerve (LFCN). While often self-limited, a subset of patients develops persistent symptoms requiring interventional management. Ultrasound guidance has improved accuracy and safety in peripheral nerve blocks, but evidence from Internal Medicine-led procedures remains limited. **Methods**: We performed a retrospective, observational case series including all patients aged ≥17 years who underwent ultrasound-guided LFCN blocks at Virgen del Rocío University Hospital between 2016 and 2024. Demographic data, comorbidities, procedural details, complications, and clinical outcomes were collected. Symptomatic response and recurrence were assessed descriptively. **Results**: Eleven patients were included (10 women; median age 56 years). The most frequent comorbidities were obesity (45.5%) and type 2 diabetes mellitus (18.2%). Clinical improvement following LFCN block was achieved in 10 of 11 patients (91%). Three patients (27%) experienced recurrence, with a median time to recurrence of 24 months; two underwent a second successful block, and one showed spontaneous resolution. No major complications occurred, and only one patient developed a mild, self-limited local reaction. **Conclusions**: Ultrasound-guided LFCN block is a safe, well-tolerated, and highly effective intervention for patients with persistent meralgia paresthetica. Outcomes achieved by an Internal Medicine specialist appear consistent with previously published reports from anesthesiology settings, underscoring the value of point-of-care ultrasound as a practical and precise tool for managing neuropathic pain within Internal Medicine settings.

## 1. Introduction

Meralgia paresthetica is a clinical syndrome characterized by pain, paresthesias, burning sensations, or dysesthesias in the anterolateral aspect of the thigh, caused by compression or irritation of the lateral femoral cutaneous nerve (LFCN) [1]. The LFCN is a purely sensory nerve that originates from the dorsal divisions of the second and third lumbar nerves from the lumbar plexus. It traverses the pelvis obliquely, passes beneath or through the inguinal ligament near the anterior superior iliac spine (ASIS), and supplies sensation to the anterolateral thigh. Due to its anatomical course and considerable anatomical variability, it is particularly vulnerable to entrapment at the level of the inguinal ligament [2,3].

The pathophysiology is most commonly related to mechanical compression. Predisposing factors include obesity, pregnancy, tight clothing or belts, prolonged standing, trauma, postsurgical changes (particularly after pelvic, spinal, or hip procedures), diabetes mellitus, and other conditions associated with increased intra-abdominal pressure. In many cases, no clear precipitating factor is identified. The estimated incidence ranges from 3 to 4.3 per 10,000 person-years, although it is likely underdiagnosed due to clinical overlap with lumbar radiculopathy and other causes of neuropathic pain [4].

The condition is frequently self-limiting, with spontaneous resolution occurring within weeks to months. Initial management focuses on removing triggering factors and initiating conservative measures, including weight reduction, avoidance of compressive garments, and pharmacologic therapy with nonsteroidal anti-inflammatory drugs or neuropathic pain agents such as pregabalin, gabapentin, carbamazepine, or phenytoin. Despite these measures, a subset of patients develops persistent or severe symptoms that significantly impair quality of life, sleep, and functional status. These patients often undergo multiple consultations across different specialties—including primary care, neurology, traumatology, pain units, and rehabilitation services—sometimes without achieving adequate symptom control or even a definitive diagnosis.

Historically, surgical management—either decompression or transection of the LFCN—was traditionally considered the definitive therapeutic option for refractory cases [5]. However, surgery is invasive and not exempt from complications, including neuroma formation or persistent sensory deficits. Over the last few decades, peripheral nerve blocks have emerged as a less invasive and highly effective alternative. LFCN block provides both diagnostic and therapeutic benefits, confirming the clinical suspicion while offering rapid symptom relief.

Initially, nerve blocks were performed using anatomical landmarks, a technique limited by the considerable anatomical variability in the LFCN. The introduction of ultrasound guidance has substantially improved the accuracy of the procedure by enabling real-time visualization of the nerve, adjacent vascular structures, fascia planes, and needle trajectory. Ultrasound guidance increases procedural precision, reduces the volume of anesthetic required, enhances efficacy, and minimizes complications such as vascular puncture or ineffective infiltration [6,7,8,9,10].

In parallel, the expanding incorporation of point-of-care ultrasound (POCUS) into Internal Medicine practice has broadened the procedural capabilities of internists. With structured training in musculoskeletal and peripheral nerve ultrasound, Internal Medicine specialists are well positioned to perform targeted, minimally invasive interventions in selected patients. Ultrasound-guided LFCN block represents a technically straightforward, reproducible, and safe procedure that can be integrated into outpatient clinical practice, potentially reducing referral delays and improving access to effective treatment.

The aim of this study is to describe a case series of 11 patients with meralgia paresthetica treated with ultrasound-guided LFCN block performed by an Internal Medicine specialist. We detail the technique employed, clinical evolution, safety profile, and observed outcomes, highlighting the feasibility and potential role of this approach within Internal Medicine departments.

## 2. Materials and Methods

We conducted a retrospective observational case series including all patients aged >17 years who underwent an LFCN block performed by an Internal Medicine specialist at Virgen del Rocío University Hospital (Seville, Spain) between 1 January 2016 and 31 December 2024.

### 2.1. Patients

Patients were referred by their primary care physicians, the emergency department, or other hospital services. Among all the patients referred to our department with meralgia paresthetica, we included only those treated by two internists trained in performing ultrasound-guided blocks. The remaining patients were managed at the discretion of their attending physician. Referral to these two internists was discretionary and not based on predefined criteria.

### 2.2. Inclusion Criteria

Patients eligible for the ultrasound-guided LFCN block were required to provide written informed consent and meet the following criteria:

- A clinical presentation consistent with meralgia paresthetica lasting at least two months.
- Recent laboratory tests conducted within the past month, indicating a platelet count ≥ 50,000/µL and an INR < 1.6.

### 2.3. Exclusion Criteria

Patients were excluded from the study if they met any of the following conditions:

- Refusal to provide informed consent.
- Inability to adequately visualize the LFCN on ultrasound due to anatomical factors (e.g., obesity).
- Known hypersensitivity to any medications used in the procedure.
- Presence of a local infection or other contraindications at the injection site.
- Treatment with P2Y12 inhibitors, including clopidogrel, ticagrelor, or prasugrel.
- Ongoing anticoagulant therapy at the time of the procedure.

### 2.4. Data, Statistical Analysis and Ethical Considerations

An ad hoc database was created for the purposes of the study, complying with current data protection and confidentiality regulations, to ensure standardized data collection in accordance with Spanish data protection law.

Data collected included age, sex, medical history related to meralgia paresthetica, date of infiltration, occurrence of complications, presence of recurrence, and time to recurrence when applicable.

A descriptive study of the different variables was conducted for the statistical analysis. The qualitative variables were expressed as absolute and/or relative frequency (n) (percent compared to the total) and the quantitative variables as median and interquartile range [Q1–Q3].

The use of clinical data was performed in a fully anonymized manner and exclusively for the purposes of this study. All data were protected in accordance with Regulation (EU) 2016/679 of the European Parliament and of the Council of 27 April 2016 on the protection of natural persons with regard to the processing of personal data. This project involved no health risks for the patients included in the study. The study received approval from the Research Ethics Committee of Virgen Macarena and Virgen del Rocío University Hospitals (SICEIA-2025-002468).

### 2.5. Procedure

Prerequisites: Prior to the intervention, patients were adequately informed about the procedure and provided written informed consent. In addition, a basic blood test including coagulation profile and platelet count within the normal range was required.

Patient positioning: Patients were placed in the supine position. Clothing had to allow for free access for the ultrasound probe along the entire thigh up to the anterior superior iliac spine (ASIS), or the area remained uncovered during the procedure.

Nerve identification technique: A high-frequency linear transducer was used. The sartorius muscle was identified by first locating the superficial femoral artery in the medial aspect of the mid-thigh; the strap-shaped muscle overlying this artery corresponds to the sartorius. The probe was then moved proximally along the thigh, following the lateral border of the sartorius, until reaching the ASIS. At this level, adjacent to the lateral border of the sartorius and in continuity with the tensor fasciae latae muscle, an aponeurotic fold resembling an “eye” could be observed. Within this fold, a round, hyperechoic structure corresponding to the LFCN was identified. In some cases, proximal branching of the nerve was visualized as smaller, rounded structures in variable number.

Block technique: The transducer was placed transversely over the aponeurotic fold of the LCFN (short axis view), a 21 g × 40 mm needle was advanced under real-time ultrasound guidance into the aponeurotic fold. The needle was always inserted using an in-plane technique from the lateral side. Hydrodissection was not considered necessary and was therefore not used. As the LFCN is a superficial nerve, it is usually easy to visualize; therefore, alternative landmark-guided infiltration techniques were not required. The only precaution is to perform the injection as cranially as possible to avoid anatomical branching proximal to the block site. Once the needle was in position, the block was performed with 1 mL of triamcinolone combined with 3 mL of 3% mepivacaine. Clinical response was typically observed within a few hours. A video demonstrating identification of the aponeurosis with the LFCN contained within it, ascending proximally along the lateral border of the sartorius muscle (Video S1), and another video demonstrating ultrasound-guided block of the LFCN (Video S2) can be downloaded as Supplementary Materials.

Follow-up: All patients were contacted by telephone within three days following the procedure. In this interview, the presence of pain was assessed in all patients using the Numeric Rating Scale (NRS), a validated self-report tool that measures pain intensity on a scale from 0 (“no pain”) to 10 (“worst imaginable pain”). They were also provided with a phone number to contact our clinic in case of recurrence. Finally, at the time of the study, a telephone call was made to assess their clinical status and whether symptoms of meralgia paresthetica had recurred. The median follow-up duration of the cohort was 62.4 months.

## 3. Results

A total of 11 consecutive patients diagnosed with meralgia paresthetica were included in the study. The cohort consisted predominantly of women (10/11, 91%), with a median age of 56 years (interquartile range [IQR] 52.5–72; overall range 46–90 years). Regarding comorbidities, obesity was the most frequent associated condition (5/11, 45.5%), followed by type 2 diabetes mellitus (2/11, 18.2%). Other relevant medical histories included hypothyroidism, temporal arteritis, carpal tunnel syndrome, and chronic alcohol use. Two patients had no relevant past medical history. A detailed description of individual baseline characteristics is provided in Table A1.

Clinical Response: Symptomatic improvement following ultrasound-guided LFCN block was achieved in 10 out of 11 patients (91%). Clinical response was defined as a significant reduction in or complete resolution of neuropathic pain symptoms in the anterolateral thigh, as reported during follow-up visits. Only one patient (9%) did not experience meaningful clinical improvement after the first block.

Recurrence and Long-Term Outcomes: During follow-up, 3 of the 11 patients (27%) experienced symptom recurrence after an initial favorable response. The median time to recurrence was 24 months (range 24–36 months). Among patients with recurrence, two underwent a second ultrasound-guided block, both achieving sustained symptom resolution after the repeat procedure. One patient developed mild recurrent symptoms at 24 months, which resolved spontaneously within a few days without requiring further intervention. Median follow-up duration was 62.4 months (IQR 32.4–78.1). Notably, no patients required surgical referral during the observation period.

Safety and Complications: No major complications were observed in any patient. Specifically, there were no cases of vascular puncture, infection, persistent sensory deficit, or systemic adverse reactions. Only one patient (9%) developed a mild, self-limiting local reaction at the block site, which resolved spontaneously without treatment. No procedure-related hospital admissions were required.

Overall Effectiveness: Considering both initial response and outcomes after repeat block, when necessary, the overall therapeutic success rate was high. When including patients who responded after a second block, effective symptom control was achieved in all but one patient in the cohort. These findings suggest that ultrasound-guided LFCN block performed by a clinical specialist with experience in POCUS is associated with a high rate of clinical improvement, low recurrence requiring intervention, and an excellent safety profile.

## 4. Discussion

Ultrasound has become an essential tool in the performance of invasive procedures, as it enables real-time visualization of anatomical structures, improving both accuracy and safety [11]. Its use allows for precise identification of relevant elements such as nerves and vascular structures, thereby reducing the risk of complications associated with inadvertent injury. This is particularly important in conditions such as meralgia paresthetica, where the anatomical variability in the lateral femoral cutaneous nerve may limit the reliability of landmark-based approaches. In this context, ultrasound-guided techniques have demonstrated clear advantages over blind procedures, including higher success rates, fewer complications, and improved patient outcomes. The ability to directly visualize the target nerve facilitates more accurate anesthetic delivery, which is especially relevant in peripheral nerve blocks such as those performed for meralgia paresthetica. Additionally, these approaches support minimally invasive management strategies, avoiding more aggressive interventions while maintaining clinical effectiveness.

In this retrospective case series, ultrasound-guided block of the LFCN appeared to be a highly effective intervention for patients with persistent meralgia paresthetica refractory to conservative management. The observed clinical improvement rate of 91% is consistent with previously reported outcomes in cohorts treated with ultrasound-guided LFCN block [6,7,10,12,13], reinforcing the therapeutic value of this minimally invasive technique.

The effectiveness observed in our cohort adds to the growing body of evidence that ultrasound guidance significantly enhances procedural success compared with landmark-based techniques. Historically, blind approaches were associated with failure rates as high as 40–60%, largely attributable to the considerable anatomical variability in the LFCN in relation to the ASIS and inguinal ligament [7,14,15,16]. Ultrasound allows for direct visualization of the nerve, surrounding fascia, adjacent vascular structures, and real-time needle advancement, thereby increasing accuracy, optimizing anesthetic distribution, and reducing the likelihood of ineffective infiltration or vascular puncture. In addition, the ability to adapt the block site to individual anatomical variation likely contributes to improved reproducibility.

Beyond technical precision, ultrasound-guided block also serves a dual diagnostic and therapeutic role. A favorable response supports the clinical diagnosis of meralgia paresthetica, particularly in cases where lumbar radiculopathy or other neuropathic syndromes are part of the differential diagnosis. This diagnostic confirmation may help avoid unnecessary imaging studies, specialist referrals, or prolonged empirical pharmacologic treatment.

Our findings indicate that ultrasound-guided LFCN block can be safely and effectively performed by Internal Medicine physicians with appropriate training in point-of-care ultrasound (POCUS). While these procedures have traditionally been carried out by radiologists or anesthesiologists, the broader adoption of POCUS has facilitated their incorporation into routine practice across other specialties. In our series, the clinical outcomes achieved were comparable to those reported in anesthesiology, pain management, and interventional radiology settings [6,7], supporting the reliability of this approach beyond its conventional domains. Real-time ultrasound guidance not only improves procedural accuracy and safety but also provides an effective framework for skill acquisition, allowing clinicians to develop and refine interventional techniques in a controlled and reproducible manner.

This paradigm shift also has organizational implications, as it supports the development of structured procedural models within Internal Medicine. The establishment of dedicated units for ultrasound-guided interventions may enhance access to minimally invasive treatments, enable earlier management of conditions such as meralgia paresthetica, and reduce delays associated with specialist referral. Ultimately, this approach may contribute to more efficient use of healthcare resources while maintaining high standards of patient care [17].

Recurrence occurred in 27% of patients, a rate comparable to that described in long-term follow-up studies [6,13]. Importantly, repeat infiltration proved effective in cases requiring additional treatment, suggesting that recurrence does not necessarily imply treatment failure but rather reflects the chronic or relapsing nature of the underlying compressive mechanism in certain individuals. Furthermore, one patient experienced spontaneous resolution of mild recurrent symptoms, reinforcing the dynamic and sometimes self-limited course of the condition.

The safety profile observed in this series was excellent. No major complications occurred, and only one patient developed a mild, self-limited local reaction. These findings are in line with the favorable safety data reported in the literature for ultrasound-guided peripheral nerve blocks [7,8,9,18,19,20]. The absence of significant adverse events supports the feasibility of performing this procedure in an outpatient setting when appropriate aseptic technique and operator training are ensured.

This study has several limitations that should be acknowledged. First, its retrospective design inherently limits the ability to establish causal relationships and introduces potential selection and information biases. Second, the small sample size (n = 11) restricts the generalizability of the findings and precludes robust statistical analysis or subgroup comparisons. Third, the absence of a control group prevents direct comparison with alternative approaches, such as landmark-based techniques or exclusive pharmacological management, limiting the strength of the conclusions regarding relative efficacy. In addition, although clinical outcomes were systematically assessed during follow-up, pain evaluation was not based on standardized, prospectively collected measures at predefined time points, which may introduce variability in outcome reporting; therefore, since the NRS pain scale was recorded only post-procedure, we are unable to compare it with baseline values, and therefore it remains largely qualitative. Furthermore, the procedures were performed by only two physicians with specific expertise in POCUS, which may limit the external validity of the results, as outcomes could differ depending on operator experience. Finally, despite the relatively long follow-up period, the retrospective nature of the study and reliance on telephone-based assessments may have led to underreporting of minor complications or symptom fluctuations over time. These limitations should be considered when interpreting the results, and highlight the need for larger, prospective, controlled studies with standardized outcome measures. Therefore, the findings should be interpreted as hypothesis-generating rather than definitive evidence.

Future prospective studies with standardized pain assessment tools, larger cohorts, and cost-effectiveness analyses would further clarify the role of ultrasound-guided LFCN block within Internal Medicine practice. Comparative studies evaluating early interventional management versus prolonged pharmacologic treatment may also help define optimal timing of the procedure in the therapeutic algorithm.

## 5. Conclusions

In conclusion, ultrasound-guided block of the lateral femoral cutaneous nerve performed by a clinician may represent a safe, minimally invasive, and effective therapeutic option for patients with persistent meralgia paresthetica. Given the small number of cases, our series can be considered an observational study suggesting a procedure with a high rate of symptomatic improvement, low recurrence requiring reintervention, and an excellent safety profile.

Beyond its therapeutic efficacy, this study supports the feasibility of performing ultrasound-guided LFCN block within the scope of Internal Medicine practice when conducted by a clinician experienced in POCUS. The integration of interventional ultrasound techniques into Internal Medicine may facilitate earlier treatment, reduce referral delays, and enhance access to effective symptom control in selected patients.

Although larger prospective studies are needed to confirm these findings and further define long-term outcomes, our results suggest that ultrasound-guided LFCN block is a reproducible and clinically valuable procedure that may expand the procedural capabilities of Internal Medicine while maintaining high standards of safety and patient-centered care.

## Data Availability

The data are not publicly available due to privacy or ethical restrictions but are available from the corresponding author upon reasonable request.

## Supplementary Materials

The following supporting information can be downloaded at: https://www.mdpi.com/article/10.3390/jcm15083094/s1; Video S1: Video demonstrating identification of the aponeurosis with the LFCN contained within it, ascending proximally along the lateral border of the sartorius muscle. Video S2: Video demonstrating ultrasound-guided block of the LFCN.

## Abbreviations

The following abbreviations are used in this manuscript:

LFCN	Lateral femoral cutaneous nerve
ASIS	Anterior superior iliac spine
POCUS	Point-of-care ultrasound

## Appendix A

**Table A1:** Description of individual with meralgia paresthetica.

Patient	Age	Sex	Other Conditions	Response to LFCN Block	NRS Post-Procedural	Minor Complications	Recurrence	Time to Recurrence (Months)	Outcome After Second Infiltration
1	46	Female	Carpal tunnel syndrome	Yes	0	No	Yes	24	Self-limited
2	53	Female	Obesity, Type 2 diabetes	Yes	0	No	Yes	24	Successful infiltration
3	65	Male	Alcohol use	Yes	0	No	No		---
4	56	Female	Obesity	Yes	0	No	Yes	36	Successful infiltration
5	48	Female	Hypothyroidism	No	6	No	-		---
6	78	Female	Temporal arteritis	Yes	0	No	No		---
7	52	Female	No	Yes	0	No	No		---
8	68	Female	No	Yes	0	No	No		---
9	55	Female	Obesity	Yes	0	Yes	No		---
10	76	Female	Obesity, Type 2 diabetes	Yes	0	No	No		---
11	90	Female	Obesity	Yes	0	No	No		---

NRS: Numeric Rating Scale.
